# High risk and early onset of cancer in Chinese patients with Peutz-Jeghers syndrome

**DOI:** 10.3389/fonc.2022.900516

**Published:** 2022-08-03

**Authors:** Zhiqing Wang, Zhi Wang, Ying Wang, Jianhua Wu, Zonglin Yu, Chudi Chen, Junsheng Chen, Baoping Wu, Ye Chen

**Affiliations:** ^1^ Department of Gastroenterology, State Key Laboratory of Organ Failure Research, Guangdong Provincial Key Laboratory of Gastroenterology, Nanfang Hospital, Southern Medical University, Guangzhou, China; ^2^ Department of Gastroenterology, Shenzhen Hospital, Southern Medical University, Shenzhen, China; ^3^ Department of Oncology, Nanfang Hospital, Southern Medical University, Guangzhou, China

**Keywords:** cancer risk, cancer spectrum, Peutz-Jeghers syndrome, rare disease, STK11 variants

## Abstract

Peutz-Jeghers syndrome (PJS) is an autosomal dominant inherited disorder associated with a predisposition to a variety of cancers. Previous studies that have evaluated the cancer spectrum and risk of this rare disease have primarily been based on small data sets or heterogeneous cohorts from different countries. Here, we report the results of a large homogeneous cohort of Chinese PJS patients who were followed prospectively from 2006 to July 2021, and clinical data before 2006 were retrospectively collected. A total of 412 PJS patients (56.55% males) from 208 families were enrolled, contributing 12,798 person-years of follow-up. A total of 113 cancers were diagnosed in 109 patients (26.46%). The median age at the first cancer diagnosis was 40 years. In particular, patients born after the 1980s were diagnosed with cancer at an earlier median age of 30.5 years. The cumulative cancer risk was sharply increased to 30.9% at age 40 years; this high cancer risk age was 10 years earlier than that reported in previous Western studies, and increased to 76.2% at an age of 60 years. The most common cancer was gastrointestinal (GI) cancer (64.6%), in which colorectal cancer constituted a significantly larger proportional distribution (32.74%), when compared with previous investigations (11.1%−20.83%). There was some evidence that overrepresentation point variants in domain XI of *STK11* may be associated with GI cancers. Furthermore, the incidences of gynecological and lung cancers were second only to that of GI cancer in this cohort. These results may provide novel insight for justifying surveillance to detect cancers at an earlier phase to improve clinical outcomes. Furthermore, the potential *STK11* genotype-phenotype association could be the basis for future genetic counseling.

## Introduction

Peutz-Jeghers syndrome (PJS) is a rare dominantly inherited disorder clinically characterized by a triad of gastrointestinal hamartomatous polyps, mucocutaneous pigmentations, and cancer predispositions (1). PJS is caused by germline mutations in serine-threonine kinase 11 (*STK11*), a tumor suppressor gene localized on chromosome 19p13.3. Increasing evidence suggests that PJS patients have an elevated risk of developing malignant tumors at different sites, including gastrointestinal (GI) and extra-GI cancers (2–6); however, the mechanism of carcinogenesis of this disease is incompletely understood. Currently, in addition to surgery and a regular endoscopic polyp resection, no precise drug for treatment and prevention exists, and only monitoring can be used.

Because it is the most severe complication and leading cause of death, PJS-related cancer has always been a major concern for patients and affected families. There have been some assessments of the mental health of patients with PJS, showing that stress surrounding the development of a malignancy was associated with a large psychological burden (7, 8), including our previous report of Chinese PJS patients who showed signs of stress when developing cancer (mean ± SD = 17.83 ± 5.83) using the Cancer Worry Scale (9). PJS patients were extremely worried and afraid of PJS-related cancers, because this disease had a significantly higher cancer risk compared with the general population and the relative risk for any cancer was reported to vary from 9.9−18 (10). Moreover, although the cancer risks for PJS patients are age-dependent because the older the patient, the higher the risk of developing cancer, malignancies can also develop in PJS patients at a young age. To date, the youngest case of GI cancer in a PJS patient was a 7-year-old boy diagnosed with well-differentiated adenocarcinoma in the jejunum (11). In addition, the mutant *STK11* gene can exist ubiquitously in all tissues, resulting in PJS patients facing the risk of many different cancers at indeterminate time points. Although several studies have been conducted to characterize the relationships between genotype and phenotype related to cancer (3, 4, 12, 13), no consensus has been reached, making it even harder to predict cancer risk.

To estimate the risk for cancer and develop reasonable surveillance programs, PJS patients have been the focus of many studies, which have resulted in important results and advancements in clinical treatments. While surveillance programs for PJS cancers have been proposed by several clinical guidelines and recommendation (1, 6, 14, 15), the validity of such surveillance programs is difficult to verify due to the relative rarity of the condition. Different cohort studies and meta-analyses of the cancer spectrum and cancer risk of PJS have been reported, but with varied results (2, 3). These inconsistent results may be due to an insufficiency of the number of enrolled patients, different schemes and durations of follow-ups, racial heterogeneities, and/or differing environments and lifestyles. Thus, the surveillance programs for PJS cancers proposed by Western countries, regardless of success and its appropriate relevance, cannot be adopted unconditionally for Chinese PJS patients. Moreover, with the existing introduction of surveillance programs tailored towards PJS patients and the increasing attention and comprehension of physicians and patients to this disease, there may be a certain impact on cancer incidence in PJS patients. Thus, an assessment of cancer risk in the Chinese population is required to provide an update.

In this study, we report the results of a large cohort of 412 homogeneous Chinese PJS patients from 208 different families. To estimate the cancer spectrum, cumulative and relative cancer risks were used to analyze the potential relationships between PJS cancers and *STK11* gene variants. The results may be valuable for patient counseling, and may provide the basis for future surveillance programs.

## Materials and Methods

### Participants

A total of 208 probands treated at Nanfang Hospital in Guangzhou from 2006 to July 2021 were included in this study. After providing verbal and written consent, their 204 PJS-affected relatives, alive or dead, were also included. When ≥ 2 individuals were affected in the same family, they were considered as familial patients. Sporadic cases were defined as having no family history of PJS, two or more hamartomatous polyps, and mucocutaneous hyperpigmentation (16, 17). Each of the 412 enrolled PJS patients originated from 29 of 34 provinces in China, which comprised an appropriate representation of the Chinese population.

All PJS patients were included in the analyses if they fulfilled the clinical diagnostic criteria^1^ recommended by the World Health Organization (1), or/and they were identified as being *STK11* gene mutation carriers. For probands who were treated at Nanfang Hospital, cancer diagnoses were reviewed and confirmed by two pathologists. For PJS patients with relatives affected with cancer, cancer diagnoses were recorded from past recollections. This project was approved for human study by the Medical Ethics Committee of the Nanfang Hospital of Southern Medical University.

### Data collection

Patients were followed prospectively between 2006 and July 2021, and clinical data from the period before 2006 were retrospectively collected. Data were obtained by performing an interview, chart review, and completing a specifically designed questionnaire. Information related to deceased patients was obtained through family recollections. The following data were collected for every patient: name, sex, date of birth, age at PJS diagnosis, family history of PJS, *STK11* gene variant status, date and cause of death, and other cancer-related information. The cancer-related information included the date of cancer diagnosis, site, histopathological type of cancer, and survival status.

We also collected information related to *STK11* variants in some cancer patients. Pathogenic germline variant screening, including Sanger direct sequencing or/and multiplex ligation-dependent probe amplification (MLPA) were performed by Nanfang hospital and other qualified institutes. In familial cases, identified variants were further tested in all available family members. All identified variants were screened against the dbSNP database to eliminate the possibility of these variants representing polymorphisms.

### Statistical analysis

Data were analyzed using SPSS 25.0 statistical software for Windows (SPSS, Chicago, IL, USA). The Kaplan-Meier method and the Cox proportional hazards model were used to estimate the cumulative cancer risks. The relative cancer risk was calculated using Poisson regression analysis. The age, sex, and tumor-specific incidence of the general Chinese population from 1989 to 2015 were derived from the National Cancer Center, Beijing, China (18–25).

## Results

### Clinical characteristics of PJS patients

All 412 PJS patients from 208 different families with a total follow-up of 12,798 person-years were included in this study (**Table 1**). The study population consisted of 233 (233/412, 56.55%) males and 179 (179/412, 43.45%) females. At the closing date (July 2021) of the study, 86 (86/412, 20.87%) patients died at a median age of 43 years (range: 19−71 years), whereas the median age of the 326 people who were still living was 30 years (range: 1−73 years). Of these, 317 (317/412, 76.94%) PJS patients had a family history from 113 (113/208, 54.33%) different families (family size range: 2−6 patients), whereas 92 (92/412, 22.33%) patients were sporadic.

**Table 1:** Baseline characteristics of 412 Chinese PJS patients from 208 different families.

Person-years of follow-up (2020)	*N* (Frequency)
Total	12798 person-years
Male	7432 person-years
Female	5366 person-years
**Gender**	
Male	233 (56.55%, 233/412)
Female	179 (43.45%, 179/412)
**Age at the end of follow-up (2020)**	
Median age (range) patients alive (n=326)	30(1-73) years
Median age (range) patients decreased (n=86)	43(19-71) years
**Family history**	
Familial PJS	317 (76.94%, 317/412)
Sporadic PJS	92 (22.33%, 92/412)
Family history unknown	3 (0.73%, 3/412)
**PJS Cancers**	
Patients with cancer	109 (26.46%, 109/412)
Cancer patients with PJS family history	100
Sporadic PJS Cancer patients	9
Cancers number	113
Patients with 2 primary cancers^a^	2
Patient with 3 primary cancers^b^	1
Median age at diagnosis of the first cancer	40 (18-71) years
PJS cancer deaths	80 (19.42%, 80/412)
Median age of cancer death	43 years
**DNA variation analysis**	
Patients screened for *STK11* variants	263 (63.83%, 263/412)
Patients with *STK11* variants	183 (69.58%, 183/263)

a One patient was diagnosed with carcinoma in the colon and cervix at the age of 23 and 30, respectively. Another patient was diagnosed with carcinoma in the small intestine and cervix at the age of 34 and 44, respectively.

b The patient was diagnosed with carcinoma in the duodenum, gallbladder, and cervix at the age of 38, 47, and 49, respectively.

Of the 412 PJS patients who were enrolled, 109 (109/412, 26.46%) were diagnosed with malignancies from 83 (83/208, 39.9%) different families. In addition, only nine cancers carrying PJS patients were sporadic vs. 100 PJS cancer patients with a family history (from 74 different families). There was a significantly higher cancer incidence in PJS familial families (74/113, 65.5%) than in sporadic cases (9/92, 9.8%) (*P* < 0.01, χ2 = 63.31). The median age at the first cancer diagnosis in the PJS cohort was 40 years. At the end of this study, 80 (80/412, 19.42%) PJS cancer patients died at a median age of 43 years.

In this cohort, 109 PJS patients suffered from 113 primary cancers. There were three cases with multiple primary cancers, which all occurred in females. The same phenomenon was observed in two PJS patients, who first developed intestinal cancers followed by cervical cancer approximately 7−10 years later. Another patient was diagnosed with carcinomas in the duodenum, gallbladder, and cervix at the ages of 38, 47, and 49 years, respectively, and survived after a timely and successful surgery. The youngest PJS cancer patient, who was a male born in 1970, was diagnosed with colon cancer at 18 years of age, and died 1 year later. The oldest PJS cancer patient was a 71-year-old male diagnosed with lung cancer in 2020, who is currently under treatment.

### Frequency and spectrum of cancers in PJS patients

PJS patients with cancer were subdivided into four groups according to the year of birth, with an interval of 20 years (**Table 2**). The majority of PJS patients with cancer were born from 1940 to 1979 (89/109, 81.7%). In addition, those (49/109, 45.0%) born between 1960 and 1979 were more likely to develop cancer than the other three age groups (*P* < 0.001). Moreover, we identified a trend that showed that with the passage of time, the age of cancer diagnosis became younger, from 49 years of age in the 1940s, shifting to an earlier age of 40 years and 30.5 years in 1960s and 1980s, respectively. Moreover, the mortality rate showed a dramatic decline, from 92.5% (37/40) in the 1940s, which decreased to 77.6% (38/49) in the 1960s and dropped to 16.7% (3/18) in the 1980s.

**Table 2:** Characteristics of 113 malignancies in 109 PJS patients.

	Distribution	Median age at diagnosis of cancer^b^ (range)	Mortality	Median age at death (range)
**Year of birth**				
1920-	2/109 (1.8%)	40, 71	2/2 (100.0%)	40, 71
1940-	40/109 (36.7%)	49 (28-69)	37/40 (92.5%)	48 (29-70)
1960-	49/109 (45.0%)	40 (18-60)	38/49 (77.6%)	41 (19-60)
1980-	18/109 (16.5%)	30.5 (24-37)	3/18 (16.7%)	29, 33, 36
**Gender**				
Male	63/109 (57.8%)	41 (18-71)	49/63 (77.8%)	43 (19-71)
Female	46/109 (42.2%)	38 (23-69)	31/46 (67.4%)	42 (27-70)
**Cancers by Origin**				
Gastrointestinal	73/113 (64.6%)	40 (18-71)	56/73 (76.7%)	45 (19-71)
Male	53/73 (72.6%)	40 (18-71)	42/53 (79.2%)	43 (19-71)
Female	20/73 (27.4%)	40 (23-64)	14/20 (70.0%)	45 (27-65)
Gynecological	15/113 (13.3%)	36 (29-49)	9/15 (60.0%)	39 (29-52)
Lung	13/113 (11.5%)	45 (24-69)	11/13 (84.6%)	45 (37-70)
Breast	9/113 (8.0%)	35 (29-56)	4/9 (44.4%)	41 (33-42)
Others ^a^	3/113 (2.7%)	33, 37, 40	1/3 (33.3%)	33
**Gastrointestinal cancer**				
Colorectum	37/113 (32.7%)	40 (18-63)	31/37 (83.8%)	42 (19-63)
Small bowel	14/113 (12.4%)	35.5 (27-66)	7/14 (50.0%)	45 (27-67)
Gastroesophageal	10/113 (8.8%)	41 (24-68)	9/10 (90.0%)	46.5 (30-69)
Liver	6/113 (5.3%)	55.5 (45-64)	5/6 (83.3%)	60 (45-65)
Pancreas	4/113 (3.5%)	56.5 (36-71)	3/4 (75.0%)	36,52,71
Gallbladder	2/113 (1.8%)	41, 47	1/2 (50.0%)	41

a Including two nasopharyngeal carcinomas, one carcinoma of the paranasal sinus;

b For fewer than four cases, the patients’ ages are shown directly.

Among the 109 PJS cancer patients, the incidence and mortality in male PJS patients (63/109, 57.8% and 49/109, 45.0%) were higher than in females (46/109, 42.2% and 31/109, 28.4%); however, no significant difference was found between different sex groups (*P* = 0.76 and 0.23, respectively). The median age at cancer diagnosis in female patients was younger than in males, which was 38 and 41 years of age, respectively (*P* = 0.06; *t* = 1.903).

For the distribution of 113 malignancies, the highest incidence of cancer was GI cancer (n = 73), accounting for 64.6%, followed by gynecological cancer (13.3%, n = 15), lung cancer (11.5%, n = 13), and breast cancer (8.0%, n = 9). The incidence of PJS GI cancer was significantly higher in males (53/73, 72.6%) than in females (20/73, 27.4%) (*P* = 0.003, χ2 = 9.301), but the median ages of diagnoses were both 40 years. Nevertheless, among 46 female PJS cancer patients, 52.2% (24/46) developed gynecological cancer or breast cancer, which was higher than for GI cancer (43.5%, 20/46). We also found that the median ages at diagnoses of gynecological (36 years) and breast (35 years) cancers were approximately 5 years earlier than that of GI cancer (40 years). In addition, the median age at the time of lung cancer diagnosis was 45 years, and 11 of 13 patients with PJS lung cancer died at a median age of 45 years.

Regarding GI cancers, the colorectum (37/113, 32.7%) was the most frequently affected organ, followed by the small bowel (14/113, 12.4%), gastro-esophagus, liver, pancreas, and gallbladder (**Table 2**). Regarding the GI cancer diagnoses, patients with malignancies in the small intestine had the youngest median age of 35 years, whereas the median diagnostic ages for colorectal cancer, gastro-esophageal cancer, pancreatic cancer, and liver cancer were 40, 41, 56.5, and 55.5 years, respectively. The mortality for each cancer was calculated and is shown in **Table 2**.

### Cumulative cancer risks

**Figure 1:**
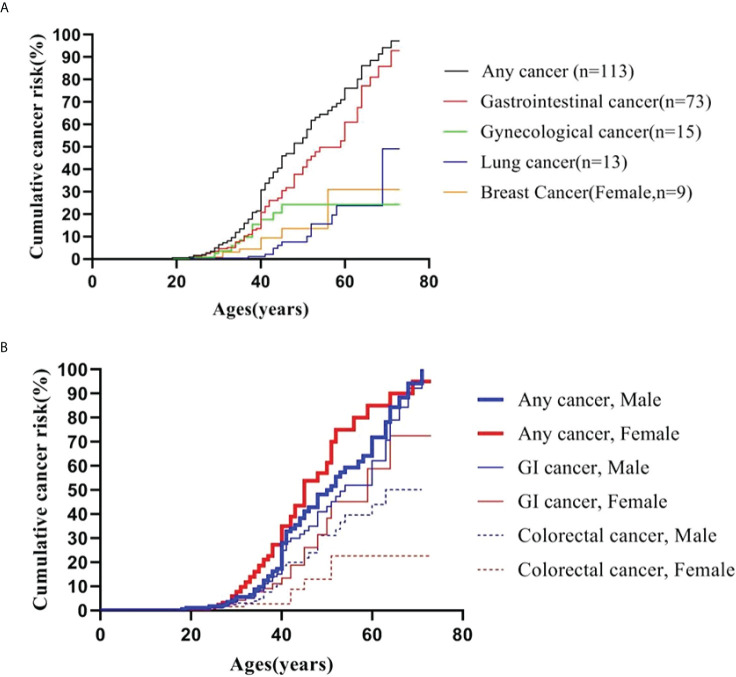
Cumulative cancer risk according to age. **(A)** Cumulative cancer risk for any cancer, gastrointestinal, gynecological, lung and breast cancer; **(B)** Gender-specific cumulative risk for any cancer, GI and colorectal cancer.

A Kaplan-Meier analysis was performed to estimate the cumulative cancer risk in PJS patients. The site-specific cumulative risks with 95% confidence interval (CI) are shown in **Table 3** and **Figure 1A**. The cumulative risk for any cancer was estimated to be 6.5% at 30 years, 30.9% at 40 years, 54.1% at 50 years, 76.2% at 60 years, and 94.2% at 70 years. The overall cumulative risk of cancer was higher in female PJS patients than in males, especially during the period from 40 years to 60 years of age (**Table 3**; **Figure 1B**); however, no significant difference was observed (log-rank test of difference: χ2 = 2.517; *P* = 0.11).

**Table 3:** Cumulative cancer risk by site and age in PJS patients.

Cancers	Cancer risk by age % (95%CI)				
	30 years	40 years	50 years	60 years	70 years
Any cancer	6.5 (3.6-9.4)	30.9 (24.0-37.7)	54.1 (45.1-63.0)	76.2 (66.7-85.7)	94.2 (87.2-100.0)
Male	5.6 (2.0-9.1)	27.9 (19.1-36.6)	49.9 (38.6-61.2)	71.8 (59.6-84.1)	94.1 (84.4-100.0)
Female	7.8 (2.9-12.6)	34.9 (23.8-46.0)	61.4 (46.4-76.4)	85.0 (70.6-99.3)	95.0 (85.7-100.0)
Gastrointestinal	4.7 (2.2-7.1)	20.8 (14.5-27.1)	37.9 (28.2-47.5)	54.8 (41.6-67.9)	78.8 (61.8-95.8)
Male	5.0 (1.6-8.4)	24.8 (16.3-33.4)	42.1 (30.4-53.8)	56.5 (42.2-70.9)	87.3 (67.4-100.0)
Female	4.2 (0.6-7.8)	13.3 (5.3-21.4)	30.2 (12.1-48.3)	53.5 (22.6-84.4)	69.0 (36.7-100.0)
Colorectum	2.4 (0.6-4.2)	13.0 (7.6-18.4)	25.3 (16.3-34.3)	38.5 (25.3-51.8)	43.7 (28.2-59.1)
Small bowel	1.6 (0.0-3.1)	6.2 (2.5-10.0)	7.6 (3.0-12.1)	—	—
Gastroesophageal	0.7 (0.0-1.7)	2.2 (0.0-4.4)	6.8 (0.8-12.8)	12.0 (0.6-23.3)	40.6 (4.0-77.2)
Liver	—	1.5 (0.0-4.5)	6.3 (0.0-13.3)	11.8 (0.0-24.2)	29.4 (5.4-53.5)
Gynecological	3.6 (0.1-7.0)	17.6 (8.3-26.8)	24.2 (11.9-36.5)	—	—
Lung	—	2.1 (0.0-4.6)	10.0 (2.6-17.4)	23.7 (9.6-37.7)	49.1 (7.3-90.9)
Breast (Female)	3.1 (0.0-6.5)	9.5 (1.6-17.4)	13.6 (2.7-24.5)	30.9 (0.0-62.4)	—

The cumulative cancer risks for GI cancer were 4.7%, 20.8%, 37.9%, 54.8%, and 78.8% at 30, 40, 50, 60, and 70 years of age, respectively. The risk was higher in males than in females, although the difference was not statistically significant (log-rank test: χ2 = 1.660; *P* = 0.19). The risks for colorectal cancer increased from 2.4% at 30 years to 13.0% at 40 years, 25.3% at 50 years, 38.5% at 60 years, and 43.7% at 70 years. The risk for colorectal cancer (30 males and seven females were affected) was significantly higher in males than in females (log-rank test: χ2 = 4.203; *P* = 0.04) (**Figure 1B**).

There were 15 PJS patients diagnosed with cancers of the gynecological system, including 11 with carcinoma of the uterine cervix, three with ovarian cancer, and one with endometrial cancer. The lifetime risk for these patients to develop gynecological cancer was 3.6% at 30 years, which increased to 17.6% at 40 years and 24.2% at 50 years. The risk of developing breast cancer (nine female patients) was 9.5% by 40 years, which increased to 30.9% at 60 years. In this series, the risks for lung cancer began to increase at an age of 50 years (10%), and subsequently reached 23.7% and 49.1% by the ages of 60 and 70 years, respectively.

### Relative cancer risks

Poisson regression analysis showed that PJS patients had a significantly higher relative risk (RR) for developing any cancer than the general Chinese population (RR: 4.7; 95% CI: 3.9−5.7; *P* < 0.001) (**Table 4**). There was no difference in the relative cancer risk (*P* = 0.83) between female PJS patients (RR: 5.6; 95% CI: 4.1−7.4) and males (RR: 4.0; 95% CI: 3.1−5.2).

**Table 4:** Relative cancer risk by site and gender in PJS patients compared to general Chinese population.

Type of Cancer	Relative Risk ^a^ (95% CI)	p Value
Any cancer	4.7 (3.9-5.7)	<0.001
Male	4.0 (3.1-5.2)	<0.001
Female	5.6 (4.1-7.4)	<0.001
Gastrointestinal	7.7 (6.0-9.7)	<0.001
Male	6.8 (5.1-9.0)	<0.001
Female	8.3 (5.1-12.7)	0.001
Gastroesophageal	2.3 (1.1-4.2)	0.15
Small bowel ^b^	37.0 (20.2-62.0)	0.03
Colorectum	16.9 (11.9-23.3)	<0.001
Liver	2.5 (0.9-5.5)	0.23
Pancreas	7.1 (1.9-18.2)	0.17
Gynecological	12.4 (7.0-20.5)	0.008
Cervix	19.3 (9.6-34.5)	0.03
Ovary	10.2 (2.1-29.8)	0.23
Uterus	2.9 (0.1-16.2)	0.56
Breast (Female)	6.0 (2.7-11.3)	0.04
Lung	2.9 (1.5-4.9)	0.05

a Compared with general Chinese population;

b Compared with data of general population from Hebei, China.

The relative risk for GI cancer was significantly increased in PJS patients compared to the general Chinese population (RR: 7.7; 95% CI: 6.0−9.7). In particular, the RR for colorectal cancer was 16.9 (*P* < 0.001) and the RR for small bowel malignancy was 37 (*P* = 0.03). However, no significant difference was found regarding the relative risk for gastro-esophageal cancer, liver cancer, or pancreatic cancer between PJS patients and the general population. Female PJS patients had a significantly increased relative risk of developing gynecological cancer compared with non-PJS patients (RR: 12.4; 95% CI: 7.0−20.5; *P* = 0.008) and the relative risk was particularly high for cervical cancer (RR: 19.3; 95% CI: 9.6−34.5; *P* = 0.03).

### Cancer-associated *STK11* gene variants

We collected 31 different *STK11* germline variants that were associated with cancer in a total of 44 index patients and/or in relatives with PJS from 35 families, including 26 (26/31, 83.87%) point variants and five (5/31, 16.13%) genomic deletions (**Table 5**). Of these *STK11* gene variations, 27 (27/31, 87.1%) were identified in familial and four (4/31, 12.9%) in sporadic cases. To our knowledge, seven (7/31, 22.58%) variants were novel: three insertion, one deletion, one synonymous variant, and two missense variants. The novel missense variants were both observed in exon 5 within the kinase domain, and *in silico* analysis revealed that both were predicted to be pathogenic (PolyPhen2: http://genetics.bwh.harvard.edu/pph2/).

**Table 5:** *STK11* gene variations of PJS patients with cancers from 35 different families.

Family	Exon	Domain	Nucleotide change	Protein change		Malignancies			
					Member	Type	Age-at-diagnose(year)	Age-at-death(year)	BirthYear
Point variants									
1F	1	I	c.138_139insGGCAA	Y49Afs*4 (Novel)	Father	Small Intestine	28	29	1982
					Grandfather	Pancreas	63	Alive	1955
2S	1	I	c.157delG	p. D53Tfs*11	Proband	Small Intestine	30	Alive	1987
3F	1	I	c.170_171insG	G58Rfs*105(Novel)	Father	Colon	40	42	1959
4F	1	I	179_180insA	p.Y60*	Grandfather	Lung	57	58	1941
5F	1	I	197_198insT	p. L67Afs*96	Father	Colon	38	38	1957
6F	Intron1	III	c.291-2 A>C	–	Father	Lung	41	41	1953
7F	2	IV	351_352insA	p.Y118Ifs*45	Grandfather	Gastric	28	30	1944
8F	3	V	403_404insTG	p.G135Vfs*27(Novel)	Mother	Cervix	29	29	1955
9F	3	VIA	c.454C>T	Q152*	Father	Colon	40	41	1958
10F	3	VIA	c.455_478dup	p.Q160_Q167dup	Father	Colon	45	46	1945
					Brother	Colon	19	19	1976
11F	4	VIA	c.509A>C	Q170P	Father	Liver	64	65	1953
					Grandfather	Pancreas	71	71	1930
12F	4	VIB	c.540delG	N181Tfs*106	Uncle	Nasopharynx	33	33	1971
					Mather	Lung	43	45	1966
					Uncle	Colon	48	49	1963
13F	Intron4	VII	c.598-2A>G	–	Proband	Uterus	30	Alive	1989
14F	5	IX	c.703A>T	p.K235*(Novel)	Mother	Gastric	40	40	1966
15F	5	IX	c.708G>A	p.V236V(Novel)	Mother	Cervix	43	46	1959
16F	5	IX	c.712A>T	p.I238F(Novel)	Uncle	Colon	39	39	1965
					Mother	Lung	52	54	1959
17F	6	X	c.787_790dup	p.E265Vfs*2	Proband	Colon	54	54	1954
18F	6	XI	c.843_844insC	p.L282Pfs*3	Father	Gall bladder	40	41	1962
19F	6	XI	c.862G>A	p.G288R	Father	Gastric	68	69	1942
20F	7	XI	c.890 G>A	p.R297K	Mother	Pancreas	50	52	1954
21F	7	XI	c.892_893insC	p.F298Sfs*20	Daugher	Small Intestine	30	30	1990
22S	7	XI	c.900delC	p.R301Gfs*35 (Novel)	Proband	Colon	36	Alive	1982
23F	7	XI	c.904 C>T	p.Q302*	Father	Colon	61	61	1960
24F	7	XI	c.904C>T	p.Q302*	Mother	Liver	45	45	1963
25F	7	XI	c.911G>C	p.R304P	Mother	Gastric	64	65	1941
26S	Intron7	XI	c.921-1G>A	–	Proband	Small Intestine	37	37	1981
27F	Intron7	XI	c.921-1G>C	–	Brother	Small Intestine	34	Alive	1985
Exonic deletions									
28F	1	c.-1114-?_290+?del		Grandfather	Liver		44		
29F	1	c.-1114-?_290+?del		Father	Colon		48	Age-at-death (year)	Birth Year
30F	1	c.-1114-?_290+?del		Father	Small		60	60	1951
					Intestine				
31F	1	c.-1114-290+?del		Mother	Breast		50	50	1960
					Aunt	Small Intestine Cervix	34 44	50	1965
32F	2		c.291-?_375+?del		Uncle	Colon	36	37	1962
					Grandfather	Colon	40	40	1935
33F	3-10		c.375-?_1365+? del		Proband	Pancreas	36	36	1973
34F	4-6		c.465-?_734+?del		Mother	Breast	40	41	1953
35S	5-7		c.598-?_863+?del		Proband	Small Intestine Gall bladder Cervix	38 47 49	Alive	1969

The types and locations of cancer-related *STK11* gene variants were evaluated. Of the 26 *STK11* germline point variants, we found that truncating variants (20/26, 76.92%) were higher than those of the missense variants (6/26, 23.08%). Intriguingly, nine (9/26, 34.62%) out of all point variants were clustered in kinase domain XI (amino acids: 277−309) and were all associated with digestive system malignancies. However, nine (9/35, 25.71%) PJS unrelated families with point variants (n = 5) or large deletions (n = 4) in the *STK11* exon one developed cancers in probands and/or the affected relatives were associated with the digestive system, as well as with lung, breast, and gynecological cancers.

## Discussion

In this study, we included 412 patients with a diagnosis of PJS, making it the largest homogeneous cohort study with a substantial period of follow-up, which amounted to 12,798 person-years. This larger sample set will provide more credible results, especially concerning the rarity of this disorder. In addition, all of the enrolled patients had the same ethnicity and were compared with the general Chinese population, who were exposed to the same environment when estimating the relative cancer risks. Another advantage of this study was that all deceased family members with a definite diagnosis of PJS were included, thereby limiting the potential survivorship bias that only patients with a less severe phenotype could be investigated.

The cancer incidence and the age of cancer onset in PJS patients have always been of particular concern. The present analysis revealed the incidences of cancer in 412 Chinese individuals with PJS, 109 (26.46%) of whom developed 113 cancers at a median age of 40 years. In 2010, the largest international systematic review of 20 cohort studies reported on 1,644 patients, 349 (21.23%) of whom developed 384 malignancies at an average age of 42 years (10). In 2016, an update on an investigation of malignant tumors associated with PJS in Japan showed a higher cancer rate and a younger diagnostic age; 186 (31.9%) of the total 583 Japanese PJS cases were identified as having at least one malignant tumor at an earlier median age of 36 years (26). In the present study, we also found that people with PJS born after the 1980s were diagnosed with cancer at an earlier median age (30.5 years), with cancer mortality significantly reduced, when compared with PJS patients born in the 1960s (40 years) and 1940s (49 years) (**Table 2**). Although the reason for this shift is uncertain, one possible explanation is that the malignant potential of PJS has been widely reported, well-recognized, and has attracted substantial attention, leading to early diagnosis and treatment.

PJS patients are thought to be prone to the development of malignancies in different organs. Therefore, it is difficult to draw definitive conclusions regarding the frequency and spectrum of malignancies from previous published studies. A meta-analysis conducted by Giardiello et al. (2) assessed 210 patients and identified 66 patients who had malignant tumors. The sites of these malignant tumors were predominantly in the GI tract, followed by the breast and pancreas. A study by van Lier et al. (10) analyzed 384 PJS-related malignant tumors, in which the most frequent site of the malignant tumors was the GI tract, followed by the breast, gynecological organs, pancreas, and lung. Moreover, Resta et al. (4) reported 36 malignant tumors in 31 out of 119 Italian PJS patients, with the most frequent site of malignancy being the GI tract, followed by the pancreas and breast. In our present analysis of 113 malignant tumors, GI cancer (64.6%) was the most common, and affected males to a significantly greater extent than females (*P* = 0.003), followed by cancer of the gynecological system, lung cancer, and breast cancer. We found that GI cancer was the most common cancer associated with PJS, in all reports; however, we found that colorectal cancer constituted a significantly larger proportional distribution of all cancers in our cohort (37/113; 32.74%), compared with previous analogous investigations and pooled data in systematic reviews [17/96 (17.7%) (3), 7/49 (14.3%) (4), 4/36 (11.1%) (6), and 80/384 (20.83%) (10) (*P*=0.01, 0.02, 0.01, and 0.009, respectively]. In 2021, the Committee of Familial Cancer of the China Anticancer Association recommended that Chinese PJS patients undergo endoscopy of the whole digestive tract every 2−3 years (27). However, these recommendations are expert opinions only, and evidence-based guidelines are presently lacking in China. Our results from the largest homogeneous cohort with substantial times of follow-up should provide help when formulating screening and surveillance protocols for Chinese PJS patients. Another difference between this study and Western reports was that the frequency of gynecological cancers was higher than that of breast cancer in females, of which carcinoma of the uterine cervix was the most common. Notably, this phenomenon was also found in a previous Japanese study (26), which may be a feature of cancer distribution among PJS patients in Asia. The discrepancy in cancer frequencies could be partially explained by racial heterogeneity, and suggested that additional importance should be attached to colorectal and cervical cancers, when formulating surveillance guidelines in China.

The potential for malignant degeneration of PJS polyps has been repeatedly discussed. A hamartoma-adenoma-carcinoma pathway has been proposed by Bosman, FT et al. (28) In spite of the small bowel being the most frequently affected region involving PJS characteristic hamartomatous polyps (29), we found fewer malignant tumors in the small bowel (n = 14) than in the colorectum (n = 37), which is consistent with other studies (2, 4, 6), but the median age at diagnosis of small bowl cancer was 35 years, which was earlier than colorectal cancer at 40 years. A possible explanation for this phenomenon might be that a large portion of PJS patients had already suffered from at least one laparotomy due to an intussusception or obstruction in the small bowel during adolescence, which could subsequently reduce the corresponding risk of cancer by surgically removing a portion of the small intestine that had large polyps or dense polyps. For example, Hinds et al. (30) reported that 68% of patients with PJS required a laparotomy for an intestinal obstruction by the age of 18 years. In contrast, the relative cancer risk in the small intestine was higher than the Chinese general population (RR: 37; *P*=0.027). Small bowel cancer is a rare cancer type that accounts for appropriately 3% of all digestive system cancers (31). It has not been well-studied because of its rarity, and we only had small bowel cancer data from Hebei Province in the general population; thus, a degree of statistical bias may be associated with the relative cancer risks. We have also learned that the pseudo-carcinomatous invasion of polyps in the small bowel may be mistaken for an invasive carcinoma (32–34), which can result in an overestimation of cancer risk. While this phenomenon was observed in approximately 10% of small bowel polyps (35), it can be distinguished from invasive carcinoma by a lack of cytological atypia, as well as negative staining of Ki67 or p53 proteins. In our present study, two pathologists identified two cases of pseudo-carcinomatous invasion of small intestine polyps, which were misdiagnosed as small bowel cancer.

**Figure 2:**
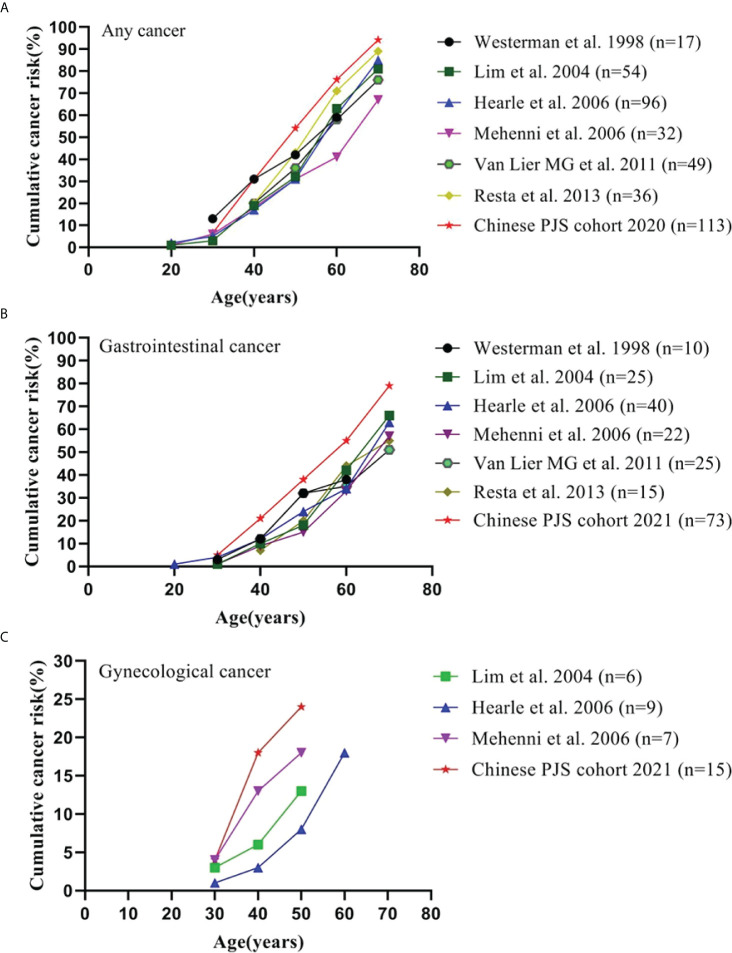
Cumulative cancer risk according to age. **(A)** Cumulative risk for any cancer; **(B)** Cumulative risk for gastrointestinal cancer; **(C)** Cumulative risk for gynecological cancer.

A highly elevated cumulative risk of developing malignancy has been established in Chinese PJS patients. The cumulative risk for overall cancer, GI cancer, and gynecological cancer in the present study together with the previous cohort studies and collaborative investigations are summarized in **Figure 2**. The cumulative risks for any cancer in our cohort were 30.9%, 54.1%, 76.2%, and 94.2% compared to that of previous studies [17%−20%, 31%−43%, 41%−71%, and 67%−89% (2, 3, 6, 13, 35)] at ages of 40, 50, 60, and 70 years, respectively (**Figure 2A**). Notably, the Chinese PJS Kaplan-Meier cumulative risk curve showed a steeper increase at 40 years of age, which was approximately 10 years earlier, when compared with that of previous Western reports (50 years of age). Such a high trend in the cumulative cancer risk in our PJS patients was also present in GI and gynecological cancers (**Figures 2B, C**). The reasons responsible for such high cumulative cancer risks in Chinese PJS patients require further investigation; however, it may be attributable to the heterogeneity associated with different races and lifestyles.

In relation to the development of malignancies, a genotype-phenotype correlation has been investigated in several studies from different countries. For correlations between the site of *STK11* genetic variants and the risk of malignancy, *STK11* gene exon 3 (36), exon 6 (13), and the C terminus and regions VIB-VIII (37) of the protein were reported to be associated with a higher cancer risk in different cohorts, respectively. Additionally, Wu et al. evaluated 38 PJS patients from 26 irrelevant pedigrees and found that individuals with missense variants had their first surgery and other symptoms significantly later than individuals with null variants (37). However, Hearle et al. (3) analyzed 419 patients with 297 *STK11* variants and found that the site and type of variant did not influence the risk of cancer. In this study, 44 PJS patients among 35 families were detected to have 31 different *STK11* germline variants that were associated with cancer. Seven variants were novel and thus extended the spectrum of known disease-causing and cancer-related *STK11* variants. For the analyses of variation types, 76.92% (20/26) cancer-related *STK11* germline point variants were truncating variants, which supported the possibility that truncating variants were associated with a more severe phenotype (17). An intriguing finding was the overrepresentation of cancer-related point variants; 34.62% (9/26) were presented within the *STK11* kinase domain XI and were all associated with digestive system malignancies. This result was similar to our previous analysis in 2014 (38), in which we identified variants in this region that were correlated with an extremely high incidence of dysplastic GFI hamartomatous polyps. As suggested by some studies, dysplasia represents a midpoint along a continuum of malignant transformation in PJS (39, 40). In the present series (it provided five more variants on the XI domain of the *STK11* gene than previously reported), we confirmed the hypothesis that variants in the XI domain of *STK11* gene should also result in a higher association with GI malignant transformation. However, future studies will be required to confirm this conclusion and should be designed to identify the associated mechanisms.

However, our analyses of the relationships between genotypes and phenotypes involved the observation that the same *STK11* genetic variant could be associated with a variable cancer status in different PJS families, or in different members of the same family, or even within a single patient. For example, the nonsense variant (Q302*) was found in two independent families: 1) a 61-year-old male with colon cancer, and 2) a 45-year-old female with liver cancer. In addition, cancer status could vary between family members sharing the same variant (e.g., N181Tfs*106), in which three PJS relatives had three different cancers, including nasopharyngeal cancer, lung cancer and colon cancer. Another female patient with a large deletion in exons 5−7 developed small bowel cancer, gallbladder cancer, and cervical cancer at 38, 47 and 49 years of age, respectively. These phenomena could be clearly confirmed by additional PJS patients with the same *STK11* exon constituting one large deletion (**Table 5**). The heterogeneity in cancer status of PJS patients with the same *STK11* variant may be partially explained by various mechanisms, in which *STK11* is involved in tumorigenesis (41–44). Another reason might be the possible influence of confounder variants in other genes, such as the activation of other different oncogenes or inactivation of tumor suppressor genes, which have been reported in many preclinical mouse models (45). Alternatively, the observed heterogeneity may be affected by environmental factors.

While these findings are important, the present study had several limitations. First, because some patients refused to conduct a pathogenic variant analysis due to privacy concerns and/or expense, the detection (263/412, 63.83%) was somewhat low in the present study. Second, the percentage of *STK11* variant carriers (183/263, 69.58%) was also relatively low. One major reason for this low percentage was because this cohort was initially recruited back in 2006, so the carrier rate was limited by the detection techniques available at the time. We plan to conduct further variant testing using more advanced detection techniques currently available to patients who have not undergone testing or for whom no genetic variant on the *STK11* gene was identified in previous testing.

## Conclusions

In conclusion, although the European Hereditary Tumor Group updated the previous clinical management recommendations of 2010 for PJS in 2021 (15), the lack of recent relevant reports means that evidence for recommendations regarding the management of PJS remains poor. It is clear from our study that cancer risks were high and sharp increased at 40 years of age rather than at the previously reported age of 50 years. Colorectal cancer constituted a significantly larger proportional distribution (32.74%) compared with previous reports (11.1%−20.83%). We further found that overrepresentation of point variants in the XI domain of the *STK11* gene was associated with GI cancer. In addition, the incidence of gynecological cancers in Chinese female PJS patients was higher than that in breast cancer, which represented another difference between Western reports. This study also provided data regarding PJS lung cancer, which was lacking in the latest guideline (15) for PJS management. Nonetheless, multicenter cohort collaborative studies will be required to avoid potential selections or publication bias in data collection, to provide superior data regarding this rare condition.

## Data availability statement

The original contributions presented in the study are included in the article/**Supplementary Material**. Further inquiries can be directed to the corresponding author.

## Ethics statement

The studies involving human participants were reviewed and approved by Nanfang Hospital of Southern Medical University. Written informed consent to participate in this study was provided by the participants’ legal guardian/next of kin.

## Author contributions

YC and ZQW: conceptualization, methodology, writing the review and editing. ZQW, ZW, and YW: data curation, investigation, writing the original draft. JW and ZY: *STK11* gene tests and supervision. CC and JC: analysis and validation of pathological features. BW: clinical examination, follow-up and clinical information collection. All authors contributed to the article and approved the submitted version.

## Funding

This work was supported by grants from the Natural Science Foundation of Guangdong Province, China (grant number 2015A030310102), Outstanding Youths Development Scheme of Nanfang Hospital, Southern Medical University (No. JQ201405) and President Foundation of Nanfang Hospital, Southern Medical University (No. 2020B007).

## Acknowledgments

The participation of all PJS family members and patients, as well as the collaboration of physicians and surgeons are gratefully acknowledged.

## Conflict of interest

The authors declare that the research was conducted in the absence of any commercial or financial relationships that could be construed as a potential conflict of interest.

## Publisher’s note

All claims expressed in this article are solely those of the authors and do not necessarily represent those of their affiliated organizations, or those of the publisher, the editors and the reviewers. Any product that may be evaluated in this article, or claim that may be made by its manufacturer, is not guaranteed or endorsed by the publisher.
